# Custom GPTs Enhancing Performance and Evidence Compared with GPT-3.5, GPT-4, and GPT-4o? A Study on the Emergency Medicine Specialist Examination

**DOI:** 10.3390/healthcare12171726

**Published:** 2024-08-30

**Authors:** Chiu-Liang Liu, Chien-Ta Ho, Tzu-Chi Wu

**Affiliations:** 1 Graduate Institute of Technology Management, National Chung-Hsing University, Taichung 402202, Taiwan; d109026203@mail.nchu.edu.tw (C.-L.L.); bruceho@nchu.edu.tw (C.-T.H.); 2 College of Health Sciences, Central Taiwan University of Science and Technology, Taichung 406053, Taiwan; 3 Department of Emergency Medicine, Show Chwan Memorial Hospital, Changhua 500009, Taiwan

**Keywords:** custom GPTs, GPT-4o, emergency medicine, evidence, knowledge

## Abstract

Given the widespread application of ChatGPT, we aim to evaluate its proficiency in the emergency medicine specialty written examination. Additionally, we compare the performance of GPT-3.5, GPT-4, GPTs, and GPT-4o. The research seeks to ascertain whether custom GPTs possess the essential capabilities and access to knowledge bases necessary for providing accurate information, and to explore the effectiveness and potential of personalized knowledge bases in supporting the education of medical residents. We evaluated the performance of ChatGPT-3.5, GPT-4, custom GPTs, and GPT-4o on the Emergency Medicine Specialist Examination in Taiwan. Two hundred single-choice exam questions were provided to these AI models, and their responses were recorded. Correct rates were compared among the four models, and the McNemar test was applied to paired model data to determine if there were significant changes in performance. Out of 200 questions, GPT-3.5, GPT-4, custom GPTs, and GPT-4o correctly answered 77, 105, 119, and 138 questions, respectively. GPT-4o demonstrated the highest performance, significantly better than GPT-4, which, in turn, outperformed GPT-3.5, while custom GPTs exhibited superior performance compared to GPT-4 but inferior performance compared to GPT-4o, with all *p* < 0.05. In the emergency medicine specialty written exam, our findings highlight the value and potential of large language models (LLMs), and highlight their strengths and limitations, especially in question types and image-inclusion capabilities. Not only do GPT-4o and custom GPTs facilitate exam preparation, but they also elevate the evidence level in responses and source accuracy, demonstrating significant potential to transform educational frameworks and clinical practices in medicine.

## 1. Introduction

Generative AI refers to artificial intelligence systems that generate new content resembling existing examples without directly copying them. These systems produce various outputs, such as images, text, music, or videos, by discerning patterns from training data. ChatGPT, developed by OpenAI (San Francisco, CA, USA), released a preview version of its GPT-3.5 model in November 2022. It stands as a sophisticated generative AI language model meticulously trained on an extensive corpus of internet text data. This model showcases the ability to generate responses very similar to human language across a variety of topics and languages, including in response to diverse queries and prompts, including medical questions or topics. However, challenges persist in analyzing complex medical data that include images and diagrams [1]. The announcement of the ChatGPT-4 Vision (GPT-4) on 25 September 2023 introduced the integration of image input capabilities, shedding new light on the crucial medical domain of image analysis. Several studies have highlighted the remarkable capabilities of GPT-4, demonstrating its proficiency in interpreting images [2,3]. Custom GPTs are customized versions of ChatGPT, based on GPT-4, that users can tailor for specific tasks or topics by combining instructions, knowledge, and capabilities [4]. The ‘Knowledge’ function and ‘Instructions’ section embedded within ChatGPT offer significant opportunities for fields that prioritize the accuracy of data, such as medicine [5].

The ‘Knowledge’ function grants the model access to a comprehensive repository of information and facilitates the development and customization of ChatGPT variants that amalgamate directives, supplemental knowledge, and a range of skills, thereby substantially enhancing their applicability. Users can upload documents infused with domain-specific expertise into the ‘Knowledge’ segment, a sophisticated feature that ensures ChatGPT provides precise and insightful responses to a wide range of queries by prioritizing learning from data deemed important by the user. In the medical domain, the need for high-quality evidence and data accuracy is paramount, making the knowledge function essential. Furthermore, the ‘Instructions’ section allows for the specification of detailed information, procedural guidelines, and critical annotations, enabling custom GPTs to engage with information sources with heightened precision. In May 2023, OpenAI held an online press conference to announce the launch of its latest model, “GPT-4o”. This advanced model is designed to excel in linguistic, textual, and visual reasoning tasks. Notably, GPT-4o is smarter, more user-friendly, and capable of evaluating user emotions, making it a significant leap forward in AI technology. GPT-4o is an advanced version of GPT-4, improving GPT-4’s capabilities and performing inference through any input combination of text, images, and audio. The features and differences among the four models above are listed in Table 1.

ChatGPT has been demonstrated to complete exams from various medical domains and has applications in medical settings, such as the Medical Licensing Exam for graduates, the United States Medical Licensing Examination, and the Otolaryngology Board Certification Examination; in emergency medicine, it has also been utilized to correctly perform mass casualty incident triage, albeit with mixed results [7,14,15,16]. Many studies suggest that even ChatGPT-4 still cannot pass relevant specialist medical exams, and the credibility and sources of the provided content are often questioned [17,18]. Thus far, no research has been conducted using ChatGPT to evaluate the official emergency medicine specialty exam, and the capabilities of LLMs in the emergency medicine domain remain unknown. Additionally, no studies have tested the effectiveness of ChatGPT’s personal ‘Knowledge’ and ‘Instructions’ functions, also known as custom GPTs. Masters et al. proposed techniques for using custom GPTs but did not provide specific experimental data to support the effectiveness of these techniques [4]. Additionally, many current studies have limitations due to differences in the types of questions or the nature of the data, which may affect the outcomes of subsequent applications. Some studies focus only on specific types of images, such as EKGs, or target specific question types or medical specialty fields [7,19,20]. Others have only conducted preliminary explorations in multimodal data fusion without in-depth analysis of its effectiveness in practical clinical applications [21]. Therefore, caution is needed when applying these techniques, and more empirical research is required to verify their effectiveness.

The goals of this study are twofold: (1) to assess the proficiency of ChatGPT in the emergency medicine specialty written examination; and (2) to compare the performance of different question-solving abilities and image recognition between version 3.5 and the more advanced GPT-4 and GPT-4o models, as well as with custom GPTs that have been enhanced with additional knowledge and instructional capabilities. This comparison aims to determine whether these models possess the necessary capabilities and access to knowledge bases required to provide accurate information. Our research addresses the following gaps:

(1) Comprehensive evaluation of multiple question types: This study encompasses various question types in emergency medicine specialty exams, including clinical knowledge questions, diagnostic questions, image-based questions, and situational application questions. This comprehensive evaluation offers a more complete understanding of AI models’ performance in complex emergency scenarios.
(2) Multi-model comparative analysis: We evaluate GPT-3.5, GPT-4, custom GPTs, and GPT-4o. This multi-model comparative analysis fills the existing gap in understanding the performance differences among various AI models on the same tasks, providing important insights for selecting the most suitable AI model for specific medical tasks.
(3) Empirical research on custom GPTs: The study provides specific experimental data to verify the effectiveness of custom GPTs. This offers valuable references for future applications, exploring the effectiveness and potential of personalized knowledge bases as an educational tool for medical residents.
(4) Cross-specialty validation: Emergency medicine is a complex discipline that demands interdisciplinary knowledge and rapid decision-making. This study provides important insights into the application of AI in the diverse medical field.

## 2. Materials and Methods

In this study, we evaluated the performance of AI models developed by OpenAI: GPT-3.5, GPT-4, custom GPTs, and GPT-4o. To enhance the consistency of the study, we used the same prompt for all models, except for Custom GPTs:

“As an emergency physician, you are undergoing a written examination for specialty certification. Please select the most appropriate option from the four given choices according to the scenario”.

In custom GPTs, we employed the “Instructions” field to guide and specify the requirements for responses. This approach integrates the concept of evidence levels.

**Figure 1:**
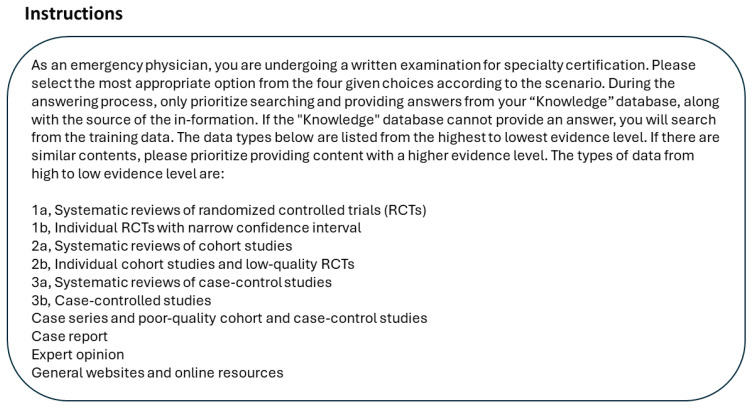
Settings for Instructions.

The settings for Instructions are as follows (Figure 1):

In addition, within our “Knowledge” database of custom GPTs, we uploaded aggregated files compiled during the residency period of emergency medicine physicians, typically sourced from reports presented at meetings, such as book reading gatherings. These contents are usually based on textbooks designated for examination preparation, such as *Tintinalli’s Emergency Medicine: A Comprehensive Study Guide*, which is one of the designated textbooks in Taiwan [22]. Due to the examination requirements, we prioritized searching the files provided by users in our instructions.

### 2.1. Data Collection

Our evaluation was conducted using 200 multiple-choice questions from the 2023 Emergency Medicine Specialist Examination, which took place on 6 May 2023, in Taiwan. These questions, primarily presented in Traditional Chinese with proprietary terms translated into English, each contained four answer options with only one correct choice. They were obtained from the official website of the Taiwan Society of Emergency Medicine “https://www.sem.org.tw/ExamRegister/PastExam (accessed on 1 April 2024)”. We manually inputted both the question text and images into ChatGPT-3.5, GPT-4, custom GPTs, and GPT-4o. To maintain consistency with other experiments, this study chose not to perform any additional special processing on the models. We used GPT-3.5, GPT-4, GPT-4o models, and Custom GPTs based on “*Tintinalli’s Emergency Medicine: A Comprehensive Study Guide, 8th Edition*”. We did not split the book content into smaller chunks for easier processing and learning by the models, nor did we conduct any additional specialized training. In terms of input, we directly input the text and images (if any) of the exam questions into each model without preprocessing or prompt engineering. The models needed to handle questions in both Traditional Chinese and English without any special language support. Regarding the answer format, we did not provide specific guidelines but allowed the models to generate responses in their default manner. The scoring standard adopted a correct/incorrect binary system, not considering partial correctness or answer quality differences. For ethical considerations, we did not provide special medical ethics guidelines but relied on the models’ existing knowledge base. For image-related questions, images were directly input into models supporting multimodal input (such as GPT-4o) without additional image preprocessing or training. In terms of error handling, no special error correction mechanisms were implemented; instead, the models’ original outputs were recorded to ensure experimental consistency and reproducibility. This approach reflects the actual performance of these AI models in emergency medicine specialty exams without special optimization, providing a benchmark for future research to compare the performance of optimized models or their application in other medical specialties. All inputs underwent manual entry, and both questions and answers were independently reviewed by one emergency physician to ensure medical precision. Finally, we compiled the rates of correct answers for further analysis.

The examination questions were reviewed and systematically organized into four distinct categories by one emergency physician. The first category, type I, focused on established diseases and clinical technologies, with an emphasis on their clinical features and treatment modalities. The second category, type II, required a differential diagnosis based on the topic at hand, calling for subsequent responses that were specifically tailored to the identified characteristics, therapeutic interventions, and overall management strategies. The third category, type III, encompassed extensive scenario-based applications or exceptional circumstances, which included, but were not limited to, aeromedical services and the management of mass casualty events. The fourth category, type IV, dealt with issues requiring memorization, including diagnostic criteria, the appropriate timing for the administration of medications, and the potential associated adverse effects.

### 2.2. Data Analysis

Our study primarily used the correct answer rate as the performance metric to evaluate the models’ performance in answering 200 multiple-choice questions. The specific indicators were as follows: Correct answer rates for different types of questions (including image-based questions, Type I, Type II, Type III, and Type IV questions). These indica-tors helped to comprehensively assess each model’s performance across different question types and revealed their strengths and weaknesses in specific areas. The rationale behind the performance metrics and statistical analysis further emphasizes the characteristics and challenges each model faces when handling different types of questions.

The variation in correct response rates, as well as the performance across different types of questions for ChatGPT among four large language models (LLMs)—specifically between the pairs (GPT-3.5 and GPT-4), (GPT-4 and custom GPTs), (custom GPTs and GPT-4o), and (GPT-4 and GPT-4o)—was evaluated using the exact McNemar test. The correct response rates for different models on four different types of questions and questions containing images were also compared. These tests were conducted on a two-tailed basis, with a *p* value below 0.05 being indicative of statistical significance. The evaluation was carried out utilizing SPSS software version 25.

## 3. Results

Within a set of 200 multiple-choice questions designed for the emergency medicine specialty examination, GPT-3.5 correctly answered 77 questions, GPT-4 correctly answered 105 questions, custom GPTs correctly answered 119 questions, and GPT-4o correctly answered 138 questions, yielding scores of 38.5, 52.5, 59.5, and 69, respectively. Of the systems, only GPT-4o achieved the passing score of 60 for the emergency medicine specialty written examination. Of the 200 questions, 28 questions included images, with GPT-3.5 correctly answering 11 questions (11/28; 39.2%), GPT-4 correctly answering 14 questions (14/28; 50.0%), custom GPTs correctly answering 15 questions (15/28; 53.3%), and GPT-4o correctly answering 18 questions (18/28; 64.2%) (Table 2).

In the performance evaluation of the emergency medicine specialty exam, the differences in performance between GPT-4o, custom GPTs, GPT-4, and GPT-3.5 reveal the potential and limitations of generative AI models. The results indicate that GPT-4o performed the best, surpassing custom GPTs; custom GPTs outperformed GPT-4, while GPT-4 was better than GPT-3.5. These findings highlight the importance of multimodal data fusion technology and specialized training in enhancing the performance of generative AI models.

Regarding the differences across all question types, GPT-4o outperformed GPT-4 (*p* < 0.05), which, in turn, outperformed GPT-3.5 (*p* < 0.05). Similarly, custom GPTs demonstrated superior performance compared to GPT-4 (*p* < 0.05), but still fell short of GPT-4o’s performance. In questions involving photographs, there was no significant difference in the capabilities of GPT-3.5, GPT-4, custom GPTs, and GPT-4o. Additionally, among the four types of questions, in Type I questions, GPT-3.5 correctly answered 36 questions (36.4%), GPT-4 correctly answered 52 questions (52.5%), custom GPTs correctly answered 58 questions (58.6%), and GPT-4o correctly answered 69 questions (69.7%). GPT-4 showed significant improvement over GPT-3.5 in handling Type I questions, while GPT-4o showed significant improvement over GPT-4 in handling both Type I and Type II questions. However, no statistically significant differences were observed in the other two question types. Conversely, custom GPTs did not achieve statistically significant differences compared to GPT-4 across all four question types (Table 3). The details about the approach for scripts, responses, and results can be found in the Supplementary Materials.

## 4. Discussion

The progression from GPT-3.5 to GPT-4, and from GPT-4 to custom GPTs and GPT-4o, has nearly elevated their scores to the passing mark of 60%, which GPT-4o has surpassed. Such findings are consistent with earlier studies that have shown more promising results for national medical licensing exams, while performances on specialist-level examinations tend to average around 60% or lower. Specialty exams demand a deeper understanding of particular medical fields than what is required for foundational tests. These examinations feature more intricate clinical scenarios that call for superior reasoning abilities and the skill to integrate information from multiple sources. Although ChatGPT is adept at comprehending context and formulating responses accordingly, it may have struggled with the complexities inherent in these specialized scenarios, potentially diminishing its success rate [23,24,25].

So far, image-based questions still pose a challenge for all models. In our study, although GPT-4o exhibited the best performance in image-based questions, GPT-4, custom GPTs, and GPT-4o did not show significant progress in correctly answering questions involving images, including those requiring the interpretation of electrocardiograms, which they failed to interpret accurately in some cases. We believe this limitation is not solely due to insufficient image recognition capabilities, but also because many question images serve a supplementary role in diagnosis. This means the clinical context provided in some questions is sufficient for ChatGPT to select the correct answer.

For Type I questions, GPT-4’s performance was noticeably superior to that of GPT-3.5, just as GPT-4o’s performance was superior to that of GPT-4, likely owing to later versions’ significantly improved ability to understand context and generate relevant responses. Custom GPTs also showed nearly significant improvements in Type I questions, indicating that targeted adjustments and training can positively impact performance in specific question types. In contrast, performance differences among models in Type III and Type IV questions were not significant, implying that these question types either present relatively lower challenges to the models or do not sufficiently reflect the capability differences among them.

We saw relevant ability enhancement and demonstration from GPT-4 to GPT-4o in this process. The results for Type I questions primarily focus on known diseases and clinical techniques, emphasizing their clinical features and treatment methods. Type II questions typically require an additional layer of thought, demanding a diagnosis based on the given context before responding. GPT-4o significantly outperformed other models, particularly GPT-4, in Type I and Type II questions, demonstrating its strong capabilities and progress in text comprehension and reasoning. This indicates that GPT-4o shows a significant advantage in handling questions related to known diseases and clinical techniques, likely due to its improved ability to understand context and generate relevant responses.

Custom GPTs, which combine text and image data by integrating multimodal data with GPT, allow the model to better understand emergency situations. For instance, it can reference textual descriptions from emergency guidelines alongside relevant medical images to make accurate judgments. Multimodal data fusion enables the model to communicate between various sources, thereby enhancing situational awareness and understanding. For image-based questions, GPT-4o converts image information into diagnostic suggestions or operational steps, improving response accuracy. To further enhance the accuracy of generative AI responses, a combination of prompt engineering, fine-tuning, and Retrieval-Augmented Generation (RAG) can be employed in the future. Prompt engineering involves designing clear questions and providing background information, while fine-tuning entails targeted training with high-quality data. RAG retrieves relevant information before generating responses. These methods complement each other, collectively improving the quality of AI responses. Our study verifies this through the use of custom GPTs within ChatGPT, demonstrating enhanced response effectiveness of GPT-4 and showing potential applications in various professional fields.

In the “Instructions” field, the chain-of-thought mechanism is commonly used to design a structured thought process that guides the AI model through specific steps for analysis and response. For example, the AI might first list the key information and symptoms from the question, then propose several possible diagnostic hypotheses, analyze the likelihood of each hypothesis while providing supporting or opposing evidence, and, finally, arrive at the most probable diagnosis or treatment plan. However, to maintain consistency in experimental results, the chain-of-thought mechanism was not implemented in this study; instead, a textbook-based approach was utilized.

### 4.1. Implementations

In Custom GPTs, we utilized “Instructions” to guide and specify the response requirements, combined with the concept of evidence levels. Because custom GPTs are based on GPT-4, their performance, although inferior to GPT-4o, still shows potential. If, in the future, they are based on GPT-4o, the advancements in custom GPTs could grow in importance in the realm of medical education across three essential dimensions. Firstly, as a supportive tool, custom GPTs significantly enhance the accuracy of the information used in the exam preparation process for medical residents. By uploading personalized notes and materials, along with the latest guidelines or protocols for managing local infectious diseases, custom GPTs are expected to access and utilize evidence-based information more adeptly than their predecessors, such as GPT-4. Previous studies have noted that ChatGPT often cited outdated articles or articles with low levels of evidence [26]. This issue has been addressed in subsequent GPT versions through tailored instruction configurations (e.g., only prioritizing searches and providing answers from the ‘Knowledge’ database). Custom GPTs can be fine-tuned to prioritize and retrieve information from the most credible sources, ensuring that the medical advice dispensed is both accurate and trustworthy.

Moreover, custom GPTs and similar AI technologies are emerging as formidable forces in promoting continuous learning among healthcare professionals, transcending the traditional educational paradigms. For example, with the introduction of new guidelines, medical staff have the capability to upload these documents and partake in a dynamic learning experience via interactive Q&A sessions with a chatbot.

Finally, custom GPTs possess the potential to radically transform conventional textbook learning methodologies. Through strategic collaborations between publishing entities and GPT technologies, subscribers or buyers could obtain access to customized LLMs. These models, by incorporating content from GPT-4 along with specialized professional insights, aim to facilitate a more efficient interaction with dependable information for emergency medical personnel, surpassing the efficiency of traditional textbook study techniques. This innovative approach is intended to augment the learning experience by ensuring access to the most current, evidence-based content in an exceptionally effective manner. However, custom GPTs still carry inherent risks, including the potential for data security breaches, such as information theft, thereby making it advisable to refrain from uploading sensitive information. Moreover, the possibility of malicious use of custom GPTs introduces privacy and security risks to users, underscoring the need for effective mitigations and defenses in future studies [4,27].

### 4.2. Limitation

This study demonstrates that the latest GPT-4o model has made significant progress in handling professional examination questions. However, our study does have certain limitations. Firstly, as previous research has indicated, the language of input can significantly affect outcomes. Since the questions were primarily presented in Traditional Chinese and contained specialized terms translated into English, an evaluation of the models’ cross-linguistic understanding capabilities is needed, as this may impact their comprehension and answer accuracy. Secondly, regarding the setup of the ‘Knowledge’ database, due to restrictions on file size and the quantity of data that could be uploaded, not all relevant information was included, potentially limiting the true capabilities of custom GPTs. Thirdly, we observed that ChatGPT occasionally provided different responses to the same question, raising concerns about the consistency of its performance. Moreover, some questions primarily rely on memory recall and may not fully reflect the AI models’ clinical reasoning and decision-making abilities. Additionally, the assessment may not have evaluated the AI’s handling of complex medical ethical issues. Finally, content specific to the Taiwanese medical system or cultural context could impact AI performance, particularly in understanding localized medical regulations and practices.

Future research could explore improvements in the construction of the ‘Knowledge’ database, including enhancing its content and method, particularly on GPT-4o, to enable custom GPTs to offer more specialized and higher-evidence-level content. Research could also extend to more specialties and application scenarios to comprehensively evaluate the practical effectiveness of generative AI models. Future studies could focus on refining evaluation methods, expanding application scopes, and validating and improving models to promote the widespread application of generative AI in the medical field.

## 5. Conclusions

In the emergency medicine specialty written exam, our findings highlight the useful-ness and potential of LLMs. GPT-4o outperformed custom GPTs, GPT-4, and GPT-3.5, particularly in Type I and Type II questions. Custom GPTs also demonstrated strong potential in enhancing model capabilities and credibility. GPT-4o and custom GPTs have the potential not only to facilitate exam preparation, but also to enhance the level of evidence in answers and provide answers from more accurate sources through specialized training and multimodal data fusion technology. With ongoing advancements in LLMs, alongside efforts to enhance and validate their accuracy and reliability, these models hold considerable potential to revolutionize both educational frameworks and clinical practices in medicine.

## Figures and Tables

**Table 1:** Features of the four types of models.

Model	GPT-3.5	GPT-4	GPT-4o	Custom GPTs	Ref.
Release Date	November 2022	March 2023	May 2024	January 2024	[5,6]
Key Features	- Excels in text generation and comprehension	- Accepts text or image inputs - Advanced text generation and reasoning	- Multimodal - Text or image inputs and outputs	- Tailored for specific domains with custom training	
Capabilities and Performance	- Strong in general natural language processing tasks - Struggles with more complex reasoning	- Superior in handling complex prompts and reasoning scenarios	- Outperforms in non-English language processing - High performance in integrating multiple data types and multi-step tasks	- Highly specialized in domain-specific tasks	
Related Studies	- More than 45 studies on medical licensing examination - Anesthesiology board exams - Plastic surgery board exams				[7,8]
		- Psychiatric licensing exams - Radiology board exams - Surgery board exams			[9,10]
		- Medical licensing exams - Specialized radiological exams			[11,12,13]

**Table 2:** Results of the performances of GPT-3.5, GPT-4, custom GPTs, and GPT-4o for questions in the Taiwan Emergency Medicine Specialist Examination.

Number of Questions		Correct Response Numbers (Rates %)			
		GPT-3.5	GPT-4	Custom GPTs	GPT-4o
All questions	200	77 (38.5)	105 (52.5)	119 (59.5)	138(69.0)
Images	28	11(39.2)	14 (50.0)	15 (53.5)	18 (64.2)
Type I	99	36 (36.4)	52(52.5)	58 (58.6)	69 (69.7)
Type II	67	26 (38.8)	33 (49.3)	39 (58.2)	47 (70.1)
Type III	19	12 (63.2)	13 (68.4)	13 (68.4)	13 (68.4)
Type IV	15	3 (20)	7 (46.7)	9 (60)	9 (60)

**Table 3:** Comparison of the performance of GPT-3.5, GPT-4, custom GPTs, and GPT-4o.

Number of Questions		Group Comparison (*p* Value)			
		GPT-3.5 vs. GPT-4	GPT-4 vs. Custom GPTs	Custom GPTs vs. GPT-4o	GPT-4 vs. GPT-4o
All questions	200	0.002 *	0.020 *	0.008 *	0.000 *
Images	28	0.435	0.453	0.508	0.289
Type I	99	0.017 *	0.210	0.052	0.002 *
Type II	67	0.210	0.100	0.115	0.003 *
Type III	19	1	1	1	1
Type IV	15	0.219	0.500	1	0.625

* *p* < 0.05.

## Data Availability

Information regarding the scripts, responses, and outcomes can be found in the Supplementary Materials. Other datasets used and analyzed during the current study are available from the corresponding author upon reasonable request.

## Supplementary Materials

The following supporting information can be downloaded at: https://www.mdpi.com/article/10.3390/healthcare12171726/s1, The approach for Scripts and responses.
