# Supplementary material for: Custom GPTs Enhancing Performance and Evidence Compared with GPT-3.5, GPT-4, and GPT-4o? A Study on the Emergency Medicine Specialist Examination
Source: Healthcare (Basel). 2024 Aug 30;12(17):1726. doi: 10.3390/healthcare12171726 (PMC11394718; doi:10.3390/healthcare12171726)
Supplement: Supplementary file 1 [file healthcare-12-01726-s001.zip › healthcare-3102598-supplementary.pdf]

Supplementary materials:

The approach for Scripts and responses.

- Prompt for all models, except for Custom GPTs:

“As an emergency physician, you are undergoing a written examination for specialty certification. Please select the most appropriate option from the four given choices according to the scenario.”

- For Custom GPTs

The settings for instructions are as follows:

As an emergency physician, you are undergoing a written examination for specialty certification. Please select the most appropriate option from the four given choices according to the scenario. During the answering process, only prioritize searching and providing answers from your “Knowledge” database, along with the source of the information. If the "Knowledge" database cannot provide an answer, you will search from the training data. The data types below are listed from the highest to lowest evidence level. If there are similar contents, please prioritize providing content with a higher evidence level. The types of data from high to low evidence level are:

1a, Systematic reviews of randomized controlled trials (RCTs)

1b, Individual RCTs with narrow confidence interval

2a, Systematic reviews of cohort studies

2b, Individual cohort studies and low-quality RCTs

3a, Systematic reviews of case-control studies

3b, Case-controlled studies

Case series and poor-quality cohort and case-control studies

Case report

Expert opinion

General websites and online resources

- Input the questions from the 2023 Emergency Medicine Specialist Examination, which took place on May 6, 2023, in Taiwan
- https://tsem.blob.core.windows.net/pastexamcontainer/112%E5%B9%B4%E5%BA%A6%E7%AD%86%E8%A9%A6%E8%80%83%E9%A1%8C(%E8%80%83%E5%8F%A4%E9%A1%8C%E5%85%AC%E5%91%8A).pdf

- The results are as follows: the performance of GPT-3.5, GPT-4, custom GPTs, and GPT-4o on the questions from the Taiwan Emergency Medicine Specialist Examination.

| Type | No. | Answer | GPT-3.5 | GPT-4 | Custom GPTs | GPT-4o |
|---|---|---|---|---|---|---|
| I | 1 | D | B | D | D | D |
| II | 2 | C | D | C | C | C |
| I | 3 | D | C | C | A | C |
| II | 4 | D | A | A | A | D |
| I | 5 | C | B | C | C | C |
| II | 6 | B | B | B | B | C |
| II | 7 | A、C | B | B | B | A |
| I | 8 | A | D | D | D | D |
| II | 9 | B | B | B | B | B |
| I | 10 | D | D | D | D | D |
| II | 11 | B | C | A | A | A |
| II | 12 | A | D | B | D | B |
| IV | 13 | C | C | C | C | C |
| I | 14 | C | B | B | B | C |
| III | 15 | C | C | C | C | C |
| I | 16 | D | C | C | D | C |
| I | 17 | A | A | A | A | A |
| IV | 18 | A | D | A | A | A |
| I | 19 | C | B | C | C | C |
| II | 20 | C | A | D | D | D |
| II | 21 | D | D | B | C | D |
| I | 22 | C | B | C | C | C |
| I | 23 | A | A | D | A | A |
| IV | 24 | D | D | D | D | D |
| I | 25 | C | C | A | A | A |
| IV | 26 | B | C | B | B | B |
| I | 27 | D | B | D | D | D |
| II | 28 | C | B | C | C | C |
| II | 29 | C | A | C | C | C |
| IV | 30 | C | A | A | A | A |
| III | 31 | C | C | C | B | C |
| I | 32 | A | A | B | B | B |
| II | 33 | C | C | C | C | C |

| Type | No. | Answer | GPT-3.5 | GPT-4 | Custom GPTs | GPT-4o |
|---|---|---|---|---|---|---|
| I | 34 | C | B | B | C | C |
| I | 35 | A | B | B | B | B |
| III | 36 | D | A | D | B | B |
| II | 37 | C | A | C | C | C |
| II | 38 | D | D | D | D | D |
| I | 39 | B | C | B | B | B |
| II | 40 | C | B | B | D | C |
| II | 41 | A | A | A | A | A |
| II | 42 | A | D | C | B | C |
| III | 43 | D | A | D | D | D |
| II | 44 | C | A | C | A | C |
| IV | 45 | B | D | C | D | C |
| III | 46 | A | B | A | A | A |
| II | 47 | C | C | C | C | C |
| II | 48 | D | D | D | D | D |
| I | 49 | B | D | B | A | B |
| II | 50 | C | C | C | D | C |
| II | 51 | A | C | A | A | A |
| I | 52 | B | D | C | C | B |
| II | 53 | A | B | D | A | A |
| I | 54 | D | A | D | D | D |
| I | 55 | C | A | D | C | C |
| III | 56 | B | B | C | B | B |
| II | 57 | B | B | C | C | B |
| I | 58 | D | C | C | C | C |
| II | 59 | B | D | D | B | B |
| I | 60 | D | D | D | D | D |
| I | 61 | B | C | B | B | B |
| I | 62 | A | A | A | A | D |
| I | 63 | C | C | B | B | C |
| II | 64 | C | D | C | C | A |
| II | 65 | C | C | C | C | B |
| II | 66 | C | C | C | C | C |
| II | 67 | C | B | C | C | C |
| I | 68 | C | C | D | D | C |
| I | 69 | C | D | C | C | C |

| Type | No. | Answer | GPT-3.5 | GPT-4 | Custom GPTs | GPT-4o |
|---|---|---|---|---|---|---|
| I | 70 | D | B | D | B | B |
| III | 71 | B | B | D | D | A |
| I | 72 | D | A | D | D | A |
| II | 73 | A | D | A | A | A |
| II | 74 | D | A | A | A | A |
| I | 75 | D | D | D | D | D |
| I | 76 | A | B | B | B | D |
| I | 77 | B | D | B | B | B |
| I | 78 | A | D | D | D | D |
| I | 79 | B | B | B | B | B |
| I | 80 | C | B | B | B | C |
| I | 81 | B | B | C | C | B |
| I | 82 | A | A | A | A | A |
| I | 83 | A | C | B | B | C |
| I | 84 | D | D | D | D | D |
| I | 85 | A | A | D | C | B |
| I | 86 | D | B | B | B | B |
| IV | 87 | C | D | D | D | B |
| I | 88 | C | A | A | A | A |
| I | 89 | A | B | A | D | A |
| IV | 90 | D | A | D | D | D |
| II | 91 | C | C | C | C | C |
| II | 92 | B | B | B | B | **B** |
| I | 93 | C | D | B | C | D |
| IV | 94 | A | D | A | A | C |
| III | 95 | B | D | A | B | B |
| I | 96 | D | C | D | D | D |
| III | 97 | A | A | A | A | A |
| II | 98 | C | C | C | C | C |
| II | 99 | B | B | B | B | B |
| I | 100 | D | B | B | D | B |
| II | 101 | D | D | C | A | A |
| III | 102 | C | D | C | C | C |
| I | 103 | C | C | B | B | B |
| I | 104 | C | C | C | C | C |
| I | 105 | A | C | B | B | A |

| Type | No. | Answer | GPT-3.5 | GPT-4 | Custom GPTs | GPT-4o |
|---|---|---|---|---|---|---|
| I | 106 | B | C | B | B | B |
| I | 107 | B | B | B | B | B |
| I | 108 | C | C | C | C | C |
| I | 109 | D | D | C | A | D |
| II | 110 | C | A | C | C | C |
| I | 111 | A | C | A | A | A |
| I | 112 | D | D | D | D | D |
| I | 113 | C | B | B | B | C |
| I | 114 | A | B | B | B | A |
| I | 115 | B | A | D | D | B |
| I | 116 | D | C | D | D | D |
| I | 117 | D | B | C | B | B |
| I | 118 | B | B | B | B | B |
| II | 119 | C | C | C | C | C |
| II | 120 | D | B | B | B | C |
| I | 121 | D | C | D | D | D |
| I | 122 | C | B | C | C | C |
| III | 123 | C | C | C | C | C |
| I | 124 | C | B | C | C | B |
| II | 125 | D | B | A | B | D |
| II | 126 | D | B | B | B | B |
| II | 127 | D | B | B | B | D |
| II | 128 | B | B | D | D | B |
| II | 129 | C | A | A | A | B |
| III | 130 | D | C | D | D | D |
| I | 131 | B | B | B | B | B |
| II | 132 | D | B | A | D | D |
| II | 133 | B | B | B | B | B |
| I | 134 | C | A | A | A | A |
| II | 135 | C | B | B | B | C |
| III | 136 | C | C | C | C | D |
| II | 137 | B | A | A | D | D |
| I | 138 | B | B | B | B | B |
| I | 139 | D | D | B | B | D |
| IV | 140 | C | D | C | C | C |
| I | 141 | D | C | C | D | D |

| Type | No. | Answer | GPT-3.5 | GPT-4 | Custom GPTs | GPT-4o |
|---|---|---|---|---|---|---|
| I | 142 | D | C | C | C | A |
| I | 143 | D | A | A | A | D |
| I | 144 | C | B | C | C | C |
| I | 145 | C | D | D | C | A |
| I | 146 | D | D | D | D | D |
| I | 147 | C | A | B | C | C |
| II | 148 | B | D | B | B | B |
| II | 149 | C | B | B | C | C |
| II | 150 | D | C | D | D | D |
| I | 151 | C | B | D | D | B |
| IV | 152 | A | B | B | A | A |
| I | 153 | C | C | C | C | C |
| III | 154 | A | D | D | D | D |
| III | 155 | B | B | A | A | A |
| I | 156 | C | C | C | C | C |
| I | 157 | C | B | B | B | C |
| II | 158 | B | A | D | D | B |
| III | 159 | C | C | C | C | C |
| I | 160 | C | D | C | C | C |
| II | 161 | D | C | D | D | D |
| I | 162 | A | A | A | A | A |
| I | 163 | D | A | D | D | D |
| I | 164 | C | B | C | C | C |
| II | 165 | A | B | A | A | A |
| I | 166 | C | C | C | C | C |
| II | 167 | B | B | B | B | B |
| IV | 168 | A | C | C | C | C |
| II | 169 | A | A | D | A | D |
| III | 170 | A | A | A | A | A |
| III | 171 | D | D | D | D | D |
| I | 172 | B | A | C | C | C |
| I | 173 | C | B | A | A | A |
| I | 174 | A | A | B | B | A |
| II | 175 | A | A | B | A | D |
| II | 176 | B | B | A | A | A |
| II | 177 | D | B | A | D | D |

| Type | No. | Answer | GPT-3.5 | GPT-4 | Custom GPTs | GPT-4o |
|---|---|---|---|---|---|---|
| I | 178 | B | C | C | C | A |
| IV | 179 | B | D | C | C | B |
| II | 180 | D | A | A | D | A |
| I | 181 | C | A | C | C | C |
| II | 182 | B | A | C | C | B |
| I | 183 | D | A | D | D | D |
| I | 184 | B | B | B | B | B |
| II | 185 | C | B | B | B | B |
| I | 186 | B | B | B | B | B |
| I | 187 | A | D | B | B | A |
| II | 188 | C | D | D | D | B |
| I | 189 | C | C | C | C | C |
| I | 190 | A | A | D | D | C |
| II | 191 | A | A | B | D | A |
| II | 192 | C | C | C | C | C |
| II | 193 | C | A | B | B | C |
| I | 194 | D | C | C | D | D |
| IV | 195 | B | B | C | C | C |
| IV | 196 | D | C | C | D | D |
| II | 197 | B | C | B | B | B |
| I | 198 | B | A | B | D | D |
| III | 199 | C | C | B | B | B |
| I | 200 | A | A | A | A | A |
| Correct Response Numbers | | | 77 | 105 | 119 | 138 |
